# The Role of OmpR in Bile Tolerance and Pathogenesis of Adherent-Invasive *Escherichia coli*

**DOI:** 10.3389/fmicb.2021.684473

**Published:** 2021-06-28

**Authors:** Valentina Lucchini, Adeline Sivignon, Michel Pieren, Marc Gitzinger, Sergio Lociuro, Nicolas Barnich, Christian Kemmer, Vincent Trebosc

**Affiliations:** ^1^BioVersys AG, Basel, Switzerland; ^2^Biozentrum, University of Basel, Basel, Switzerland; ^3^Université Clermont Auvergne, Inserm U1071, USC-INRAE 2018, Microbes, Intestin, Inflammation et Susceptibilité de l’Hôte (M2iSH), Clermont-Ferrand, France

**Keywords:** OmpR, AIEC, virulence, CEABAC10 mice, sodium deoxycholate, tolerance

## Abstract

Gut microbiota dysbiosis toward adherent-invasive *Escherichia coli* (AIEC) plays an important role in Crohn’s disease (CD). The OmpR transcriptional regulator is required for the AIEC LF82 prototype strain to adhere and invade intestinal epithelial cells. In this study, we explored the role of OmpR in AIEC pathogenesis using a panel of eight *Escherichia coli* strains isolated from CD patients and identified as AIEC. The deletion of *ompR* together with the implementation of two cell-based assays revealed that the role of OmpR in adhesion *in vitro* was not conserved in AIEC clinical strains. Nevertheless, we showed that OmpR was required for robust gut colonization of transgenic mice expressing human CEACAM receptors, suggesting that OmpR is involved in alternative virulence mechanisms in AIEC strains. We found that deletion of *ompR* compromised the ability of AIEC strains to cope with the stress induced by bile salts, which may be key for AIEC pathogenesis. More specifically, we demonstrated that OmpR was involved in a tolerance mechanism toward sodium deoxycholate (DOC), one of bile salts main component. We showed that the misregulation of OmpF or the loss of outer membrane integrity are not the drivers of OmpR-mediated DOC tolerance, suggesting that OmpR regulates a specific mechanism enhancing AIEC survival in the presence of DOC. In conclusion, the newly discovered role of OmpR in AIEC bile tolerance suggests that OmpR inhibition would interfere with different aspects of AIEC virulence arsenal and could be an alternative strategy for CD-treatment.

## Introduction

Crohn’s disease (CD) is a type of inflammatory bowel disease (IBD), which is a complex chronic disorder that primarily disturbs the digestive system (Crohn’s disease, 2020). CD affects more than 2.5 million individuals in Western countries and it has an increasing incidence in the newly industrialized world (Ng et al., 2017). Interactions between the altered intestinal microbiota, the abnormal mucosal immune system of the host and environmental factors contribute to the onset and progression of IBD diseases (Elhenawy et al., 2018; Chervy et al., 2020).

Adherent-invasive *E. coli* (AIEC) is a functionally distinct group of resident mucosa-associated bacteria that is enriched in CD patients (Darfeuille-Michaud, 2002). In the absence of common identifying genetic determinants, AIEC strains have been characterized by their ability to adhere and invade intestinal epithelial cells (Camprubí-Font et al., 2019; Camprubí-Font and Martinez-Medina, 2020). AIEC adhesion and invasion is mainly mediated by the type 1 pili and their adhesin FimH that bind to the glycoproteins, such as carcinoembryonic antigen-related cell adhesion molecule 6 (CEACAM6) that are enriched at the surface of ileal epithelial cells of CD patients (Boudeau et al., 2001; Barnich et al., 2007). *In vitro* studies have demonstrated that AIEC strains are able to extensively survive and replicate within monocytes-derived macrophages (Glasser et al., 2001). AIEC strains have been described as moderate to strong *in vitro* biofilm producers (Martinez-Medina et al., 2009). Altogether, these attributes potentially contribute to AIEC mediated exacerbation of CD through their enrichment in ileal mucosa leading to chronic intestinal inflammation.

Two-component signal transduction systems (TCSs) have evolved to coordinate bacterial communication with the host as an important aspect of both symbiosis and pathogenesis (Calva and Oropeza, 2006). OmpR, the transcriptional regulator of the OmpR/EnvZ TCS, was shown to be essential for FimH-mediated *in vitro* adhesion and invasion of intestinal epithelial cells in the prototype LF82 AIEC strain (Rolhion et al., 2007). These results suggest that OmpR inhibition may be an interesting approach to prevent AIEC-mediated exacerbation of CD.

The goal of the present study was to uncover the contribution of OmpR in AIEC pathogenesis using diverse clinical strains isolated from CD patients to evaluate the clinical relevance of OmpR as a drug target. We found that, despite a non-conserved OmpR-mediated *in vitro* adhesion phenotype, OmpR was required for robust AIEC colonization of CEABAC10 mouse gut. In addition, we demonstrated that OmpR plays a role in AIEC bile tolerance, which may be central for AIEC pathogenesis.

## Materials and Methods

### Bacteria Strains and MIC

Eight adherent-invasive *Escherichia coli* (AIEC) clinical isolates and the reference K12 MG1655 *Escherichia coli* strain were used in this study (Table 1). Multiple locus sequence type was determined according to the Pasteur scheme (Clermont et al., 2015). The microdilution method was used to determine the minimum inhibitory concentration (MICs) in cation-adjusted Mueller-Hinton broth (CA-MHB) according to the CLSI guidelines (CLSI, 2020). Bacteria were grown using Luria-Bertani (LB) broth or agar at 37°C unless otherwise stated.

**TABLE 1:** Description of AIEC strains selected for the study.

Strain	Isolation	ST	FimH mutations^a^	References
MG1655	/	262	WT	This study
7136	Feces from CD	400	V27A, N70S, G73R, and S78N	Dreux et al. (2013)
S136	Ileal specimen from CD	732	V27A, N70S, and S78N	This study
S179	Ileal specimen from CD	73	V27A, N70, and S78N	This study
S135	Ileal specimen from CD	519	V27A, N70S, and S78N	This study
S244	Ileal specimen from CD	4	V27A, N70S, S78N, and V163A	This study
S52	Ileal specimen from CD	64	V27A, N70S, S78N, and T158P	This study
S162	Ileal specimen from CD	127	V27A, N70S, and S78N	This study
LF31	Ileal specimen from CD	29	V27A, G66A, N70S, S78N, and A106T	Darfeuille-Michaud et al. (2004)

*^*a*^FimH mutations compared to K12 MG1655 *E. coli* strain.*

*ST: sequence type.*

### Genomic Deletion of *ompR* in AIEC Strain and Complementation

Scarless deletions of *ompR* were performed using a two-step recombination method previously described (Trebosc et al., 2016). Briefly, DNA fragments corresponding to 700-bp up- and downstream genomic regions of *ompR* were amplified by PCR using oCK647/oCK648 and oCK649/oCK650, respectively (Supplementary Table 1). The obtained fragments were cloned using NEBuilder HiFi DNA assembly (New England Biolabs) in the multiple cloning site of the *E. coli* knockout plasmid pCK452. The pCK452 plasmid is a derivative of the pVT77 plasmid where the ColE1 origin of replication has been replaced by the *E. coli* non-replicative R6K origin of replication and the IPTG regulated thymidine kinase expression cassette has been replaced by an anhydrotetracycline (aTC) inducible SceI expression cassette, corresponding to the counter selection system (Trebosc et al., 2016). The cloned knockout plasmid was transformed in *E. coli* conjugative strain MFDpir to proceed with the construction of markerless deletion in AIEC. Briefly, after conjugation, genomic plasmid integration was selected on LB agar plates containing 100 μg/ml sodium tellurite. Clones were screened for up- or downstream integration by PCR using primer oCK576, which anneals on the genome upstream of the *ompR* flanking region, and oCK354, which anneals on the plasmid. Clones containing up- and downstream plasmid integrations were transferred on LB agar plates containing 2 μg/ml aTC to select for plasmid removal from the genome. Clones were screened for gene deletion and plasmid removal by PCR using primers oCK576/oCK578 (Supplementary Table 1). The genomic gene deletions were finally confirmed by DNA sequencing (Microsynth AG, Balgach, Switzerland). The *ompR* gene was PCR amplified using oVT559/oVT331 and cloned using NEBuilder HiFi DNA assembly in the multiple cloning site of the pSEVA324 expression plasmid to construct the OmpR complementation plasmid.

### Yeast Aggregation Assay

Commercial baker’s yeast was diluted in phosphate-buffered saline (PBS) to 0.5 mg/ml. AIEC cultures incubated at 37°C in LB for 24 h without shaking were collected and diluted in PBS to OD_600_ = 5. Equal volumes (50 μL) of a fixed concentration of yeast suspension and decreasing concentrations of *E. coli* suspension were mixed in a 96-well plate (Claret et al., 2007). After 2 h, the aggregation was monitored visually and the titer was recorded as the lowest dilution of bacteria giving a positive aggregation reaction (Supplementary Figure 1).

### Adhesion Assay

The human intestinal epithelial T84 cell line was obtained from the American Type Culture Collection (ATCC^®^ CCL248). T84 cells were cultured in Dulbecco’s Modified Eagle Medium: Nutrient mixture F-12 supplemented with 2 mM L-Glutamine, 0.1 M HEPES buffer, 10% fetal bovine serum and antibiotics-antimycotics to a final concentration of 100 U/ml penicillin G, 100 μg/ml streptomycin, and 0.25 μg/ml of amphotericin B. T84 monolayers were seeded in 24-well tissue culture plates with 4 × 10^5^ cells/well and incubated for 48 h. Cell monolayers were washed 2 times with PBS and infected with AIEC bacteria for 3 h at 37°C, in an atmosphere containing 5% of CO_2_ at a multiplicity of infection (MOI) estimated at 10 bacteria per cell. For that, bacterial suspensions were prepared at a concentration of 1.6 × 10^8^ bacteria/ml in culture medium and 25 μL were added onto the cells to reach ≈4 × 10^6^ bacteria/well (=MOI 10). Cells were washed 4 times with PBS and the T84 cells were lysed with X-100 Triton 1% for 5 min at room temperature, which is a lysis treatment that does not affect bacterial cells (Supplementary Figure 3). Finally, the lysates were appropriately diluted and spread on LB agar plates and incubated at 37°C, overnight.

### MET Analysis

Bacteria were placed for 5 min on carbon-formvar copper grids and negatively stained during 30 s with Uranyless (Delta Microscopies, France). Samples were observed using a Hitachi transmission electron microscope (H-7650, Japan) at 80 kV acceleration voltage. Micrographs were made using a Hamamatsu camera placed in a side position.

### CEABAC10 Mouse Infection Model

Eight-week-old CEABAC10 male mice [heterozygous, (Chan and Stanners, 2004)] were used to perform *in vivo* experiment for testing wild-type (WT) and *ompR* deleted (Δ*ompR*) AIEC strains. Mice were housed in specific-pathogen-free conditions (21–22°C, 12:12-h light-dark cycle), with access to food and water *ad libitum*, in the animal facility of the University Clermont Auvergne (EOPS animal care facility, Clermont-Ferrand, France). This study was carried out in strict accordance with the recommendations of the Guide for the Care and Use of Laboratory Animals of the University of Clermont Auvergne (Clermont-Ferrand, France). The animal protocol was approved by the Committee for Research and Ethical Issues of the Department of Auvergne (CEMEA Auvergne; Permit Number: CEMEAA, 2018103015295515). CEABAC10 mice were treated with streptomycin in drinking water (1.5 g/L) and a low concentration of 0.5% of DSS (dextran sulfate sodium salt colitis grade, MP Biomedicals) for 3 days to disrupt normal resident bacterial intestinal microbiota and to induce a low-grade inflammation. DSS/Streptomycin was replaced by DSS 0.5% 24 h before AIEC infection. The day of infection (day 0), overnight bacterial cultures of AIEC strains were harvested by centrifugation at 2500 × *g* for 10 min. The bacterial pellet was resuspended in PBS at 1.5 × 10^10^ bacteria/ml. Mice were challenged with 3 × 10^9^ bacteria (0.2 ml), immediately after an oral administration of 0.1 ml of sodium bicarbonate at 0.2 M. At day 7 post-infection, fresh fecal pellets (≈100 mg) were collected and resuspended in PBS. After appropriate serial dilutions, AIEC bacteria were enumerated by plating on LB agar medium containing 16 μg/ml vancomycin.

### AIEC Growth on MacConkey and Drigalski Selective Agar Plates

AIEC WT, Δ*ompR*, and complemented mutants were grown for 24 h at 37°C in LB without shaking. The cultures were normalized to an OD_600_ of 1 and 10-fold serial dilutions were plated on selective MacConkey and Drigalski agar plates (Condalab) and on non-selective LB agar plates. Colony forming units (CFU) were counted after 24 h incubation at 37°C.

### Time-Kill Curves

Overnight LB cultures of AIEC WT and Δ*ompR* mutants were diluted to OD_600_ = 0.05 in 10 ml of fresh LB broth in presence of bile salts at the specified concentrations. The cultures were incubated at 37°C with shaking at 200 rpm and samples were taken at 1, 2, 4, 6, 8, and 24 h to determine the OD_600_ and the CFU/ml. Samples were plated on LB agar and CFU were counted after 24 h incubation at 37°C.

### Quantitative Real Time PCR (qRT-PCR)

The expression of *ompR*, *ompC*, *ompF*, and *mdtE* was quantified using qRT-PCR. The strains were grown in LB broth at 37°C to mid-log (OD_600_ of 0.4), late-log (OD_600_ of 1.2), or late stationary (OD_600_ of 4.5) growth phase and total RNA was extracted using a PureLink RNA minikit (Ambion) according to the manufacturer’s recommendations. Residual DNA contaminations were removed using a Turbo DNA-free kit (Ambion). Quantitative reverse transcription-PCR (qRT-PCR) was performed using a GoTaq 1-Step RT-qPCR System kit (Promega, Madison, WI, United States) on a StepOne Real-Time PCR LightCycler (Applied Biosystems, Foster City, CA, United States). The expression of the target genes was normalized to that of the *rpoD* housekeeping gene using the comparative ΔΔ*C*_*T*_ (where *C*_*T*_ is threshold cycle) method. The primers used in qRT-PCR can be found in Supplementary Table 1.

## Results

### The Role of OmpR in Adhesion Is Not Conserved in all AIEC Strains

**FIGURE 1:**
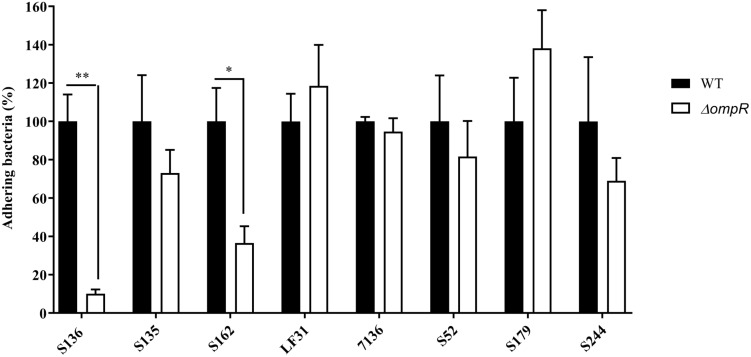
Adhesion levels of AIEC WT and Δ*ompR* mutants to intestinal epithelial cells T84. Adhesion assay was performed with T84 intestinal epithelial cells infected with WT (black bars) and Δ*ompR* mutants (white bars) at a MOI of 10 bacteria/cell for 3 h. Results are expressed in %, considering 100% as adhesion level for the WT strain (means ± SEM, 5 independent experiments). ^∗^*p* < 0.05, ^∗∗^*p* < 0.01. Unpaired *t* test.

A panel of 8 diverse AIEC clinical strains isolated from 8 different CD patients was used to evaluate the clinical relevance of OmpR as a drug target (Table 1). All the strains belonged to distinct sequence types and encoded different FimH variants, reflecting the genotypic variability of the AIEC group (Desvaux et al., 2020). Except for the strain S179, all AIEC strains belonged to the S70/N78 FimH clade, conferring higher ability to adhere to intestinal epithelial cells (Dreux et al., 2013). The non-pathogenic K12 MG1655 *E. coli* strain was included as a control strain. The *ompR* gene was deleted in all the AIEC strains using a markerless recombination method (Trebosc et al., 2016) leading to clean gene deletion. The deletion of *ompR* did not significantly affect the *in vitro* activity of standard of care antibiotics, with a maximum of one out of the 8 AIEC strains tested showing a 4-fold MIC shift (Supplementary Table 2). To evaluate the role of OmpR in AIEC adhesion properties we first used a yeast aggregation assay that allows to study FimH-mediated binding to the highly mannosylated yeast cell wall structure. The deletion of *ompR* abolished the yeast aggregation ability of 3 out of the 8 AIEC strains, namely S136, S135, and S162 (Table 2). Alternatively, we assessed the ability of the AIEC strains and their isogenic Δ*ompR* mutants to adhere to T84 intestinal epithelial cells expressing the specific CEACAM6 receptor (Dreux et al., 2013). Deletion of *ompR* significantly impaired the adhesion abilities of the S136 and S162 strains (Figure 1 and Supplementary Figure 4). Together, the results from two orthogonal bacterial adhesion methods indicate that the previously described role of OmpR in AIEC adhesion properties is not conserved in all AIEC strains.

**TABLE 2:** Yeast aggregation titer for the WT and Δ*ompR* AIEC strains.

	Yeast aggregation titer*								
	
Strain	S136	S135	S162	LF31	7136	S52	S179	S244	MG1655
WT	0.08	0.02	0.04	0.04	0.02	0.01	0.04	0.16	0.63
Δ*ompR*	>2.5	>2.5	>2.5	0.31	0.02	0.02	0.04	0.63	1.25

*The yeast aggregation titer is defined as the lowest dilution of bacteria giving a positive aggregation reaction as illustrated in Supplementary Figure 1.

Mutation in OmpR may affect the expression of regulated genes and explain the difference in adhesion properties between AIEC strains (Vidal et al., 1998). However, none of the AIEC strains studied encoded a mutation in OmpR. In addition, we sequenced the promoter of the *fimB* and *fimE* genes encoding recombinases that control type I pili expression through the inversion of the *fimS* element encoding the promoter of *fimA* (Schwan, 2011). No mutations were found in the P1 and P2 promoters of *fimB* while only the AIEC strain S162 encoded a point mutation in the P1 promoter of *fimE* (Supplementary Figure 2). Together, neither OmpR mutation nor *fimB/E* promoter mutation can explain the divergent role of OmpR in AIEC adhesion phenotype.

### OmpR Is Involved in AIEC Intestinal Colonization of CEABAC10 Mice

**FIGURE 2:**
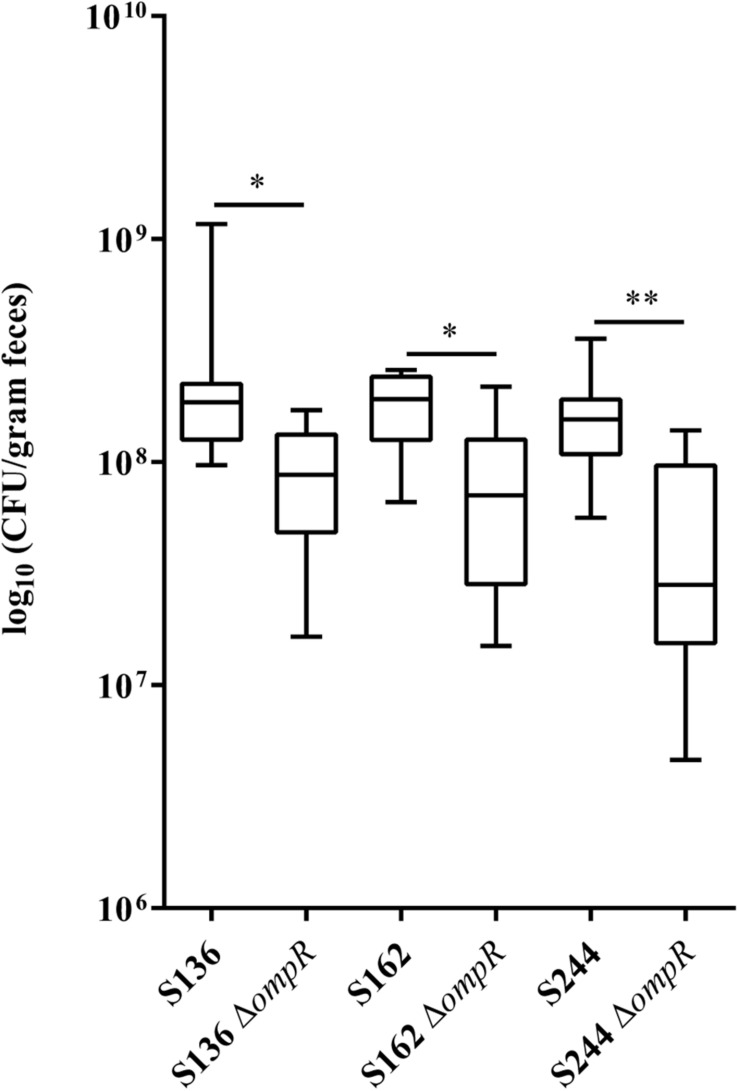
Ability of AIEC WT and Δ*ompR* mutants to colonize the intestine of CEABAC10 mice. Bacterial load in the feces of CEABAC10 mice (8 mice per group) infected with WT and Δ*ompR* AIEC strains at 7-day post-infection. The quantification of AIEC bacteria in the feces of CEABAC10 mice are expressed in CFU/gram feces. Representation in box and whiskers (Min to Max); Mann Whitney test realized between WT and *ompR* mutant groups; ^∗^*p* < 0.05 and ^∗∗^*p* < 0.01.

CEABAC10 transgenic mice were used to determine the contribution of OmpR in the ability of AIEC bacteria to colonize the gastrointestinal tract. This mouse model expresses the human CEACAM6 glycoprotein that is enriched in CD ileal epithelial cells and favors AIEC gut colonization through type 1 pili mediated recognition of mannose residues harbored by CEACAM6 (Carvalho et al., 2009). We selected two AIEC strains (S136 and S162) strongly impaired in their *in vitro* adhesion abilities in absence of *ompR* and one AIEC strain (S244) not significantly affected in its adhesion phenotype to evaluate the role of OmpR in AIEC virulence. At day 7 post-infection, the bacterial loads in the feces of CEABAC10 mice were significantly lower in the three Δ*ompR* mutants compared to their WT ancestors (Figure 2). These results indicate that OmpR is required for AIEC robust colonization of mouse gut in CD conditions. Interestingly, the AIEC strain S244, whose *in vitro* adhesion properties were not affected by *ompR* deletion, showed the most pronounced impairment in gut colonization upon loss of OmpR. The lack of correlation with epithelial cell adhesion phenotype suggests that OmpR regulates alternative virulence mechanisms that are required for AIEC pathogenesis.

### OmpR Is Involved in the Ability of AIEC Strains to Grow in the Presence of Bile Salts

**FIGURE 3:**
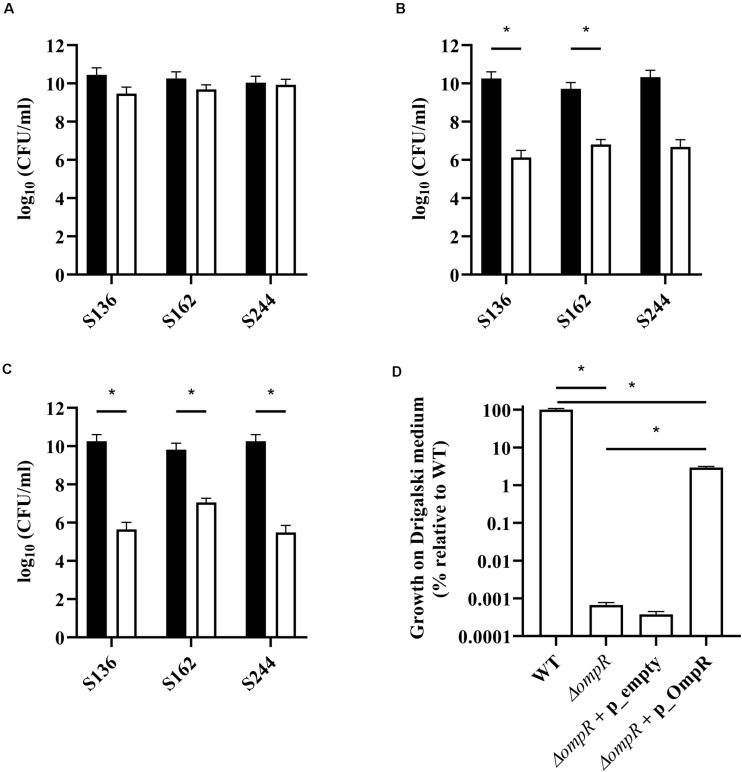
Quantification of AIEC WT and Δ*ompR* mutants in different agar media. WT (black bars) and Δ*ompR* (white bars) AIEC strains were inoculated on **(A)** LB, **(B)** MacConkey, and **(C)** Drigalski agar media and the CFU/ml were determined after overnight growth at 37°C (means ± SEM, 2 independent experiments). **(D)** The WT AIEC S244 strain and its respective Δ*ompR* mutant complemented with an empty plasmid (Δ*ompR* + p_empty) or a plasmid expressing OmpR (Δ*ompR* + p_OmpR) were inoculated on Drigalski agar and the CFU/ml were determined after overnight growth at 37°C (means ± SEM, 3 technical replicates). *p < 0.05, unpaired *t* test.

**FIGURE 4:**
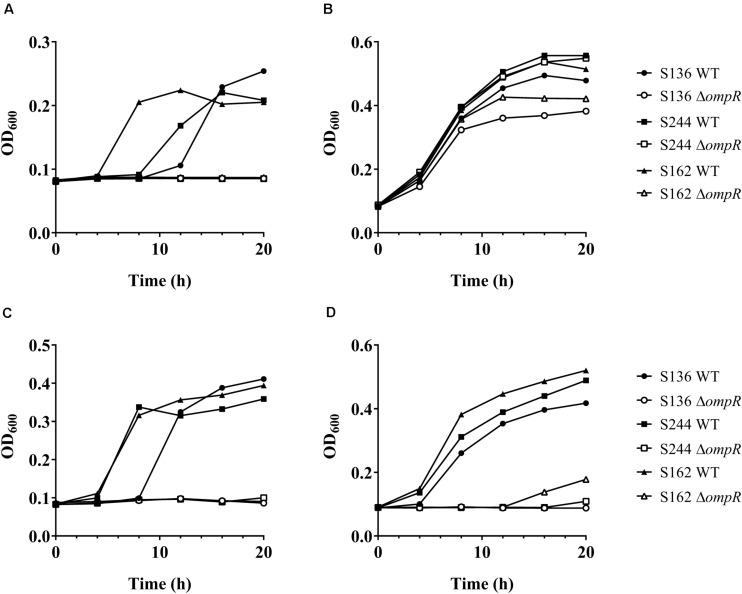
Growth of AIEC WT and Δ*ompR* mutants in presence of bile salts. The WT (filled symbols) AIEC strains S136 (circle), S244 (square), and S162 (triangle) and their Δ*ompR* mutants (empty symbols) were grown in LB supplemented with **(A)** 0.5% bile salts, **(B)** 0.1% sodium cholate, **(C)** 0.1% DOC, and **(D)** 0.1% chenodeoxycholate. Data representative of at least two independent experiments.

Bile salts and their detergent properties represent one of the major bacterial selecting agents in the gut, suggesting that adaptation to bile induced stress plays an important role in AIEC pathogenesis (Urdaneta and Casadesús, 2017; Delmas et al., 2019). Interestingly, the Δ*ompR* mutants showed impaired growth in MacConkey and Drigalski media, which both contain bile salts as a selective agent, whereas growth on selective medium was partially restored in a Δ*ompR* complemented strain (Figure 3). The two most abundant bile salts are sodium cholate and sodium deoxycholate (DOC), which are found at equivalent concentrations in bile while DOC is prevalent in the feces due to the biotransformation by the gut microbiota (Ridlon et al., 2006; Pavlidis et al., 2015). Physiological concentrations of bile salts and DOC specifically inhibited the growth of the Δ*ompR* AIEC mutants, while the growth of the Δ*ompR* AIEC mutants was still observed in the presence of the same concentration of sodium cholate (Figure 4). To note, increased concentrations of cholate may lead to similar growth inhibition than the one observed with DOC, however, this would be less relevant in the context of AIEC physiological niche where DOC is prevalent. In addition, a DOC derivative (chenodeoxycholate) exerted the same growth inhibition as DOC on the Δ*ompR* AIEC mutants. These data indicate that OmpR is involved in the regulation of DOC stress response in AIEC strains, which may be a driver of the attenuated virulence observed in the AIEC Δ*ompR* mutants.

### OmpR Is Involved in AIEC Sodium Deoxycholate Tolerance

**FIGURE 5:**
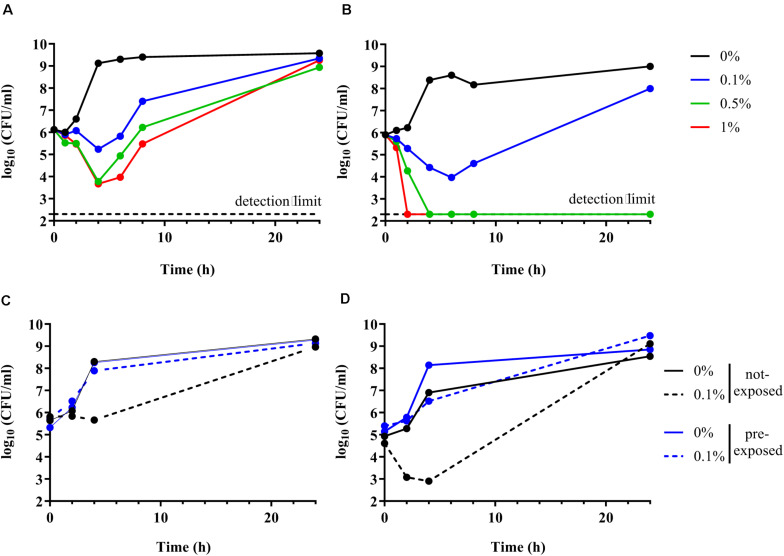
Time kill curve of AIEC WT and Δ*ompR* mutant in presence of DOC. **(A,B)** The WT AIEC strain S136 **(A)** and its Δ*ompR* mutant **(B)** were grown in LB without DOC (black line) or with 0.1% (blue line), 0.5% (green line), and 1% DOC (red line). **(C,D)** The S136 WT **(C)** and Δ*ompR* mutant **(D)** recovered after 24 h of an LB culture without DOC (not-exposed, black line) and with 0.1% DOC (pre-exposed, blue line) (see Figures 5A,B) were grown again in LB without DOC (filled line) and with 0.1% DOC (dotted line). Data representative of at least two independent experiments.

Time kill curve experiments were performed to confirm the inability of the Δ*ompR* AIEC mutants to respond to the stress induced by DOC. Unexpectedly, both the AIEC WT and the Δ*ompR* mutant showed initial CFU reduction in the presence of DOC followed by a regrowth after 6 h (Figures 5A,B). These data indicate that DOC is bactericidal against AIEC strains independently from the presence of a functional OmpR. Nevertheless, the killing rate against the Δ*ompR* mutant was more pronounced than against the WT, allowing only the WT to regrow in the presence of 0.5 and 1% DOC. These data indicate that OmpR is involved in the regulation of AIEC DOC tolerance mechanisms, since bacterial tolerance is characterized by a reduced killing rate (Balaban et al., 2019). In addition, we investigated if the regrowth was due to decay in the concentration of DOC or to the acquisition of resistance. We evaluated the ability of the WT and Δ*ompR* mutant recovered after regrowth (at 24 h) to grow again in the presence of the same DOC concentration. DOC-mediated killing was not observed anymore on DOC pre-exposed strains for both the WT and the Δ*ompR* mutant (Figures 5C,D). A passage on non-selective media of the pre-exposed strains led to the same results (data not shown). These results indicate that the regrowth observed after DOC-mediated killing is due to the development of a stable resistance mechanism and that OmpR is not involved in the acquisition of this mechanism.

### OmpR-Mediated DOC Tolerance Is Not Due to OmpC/OmpF Porin Misregulation or Loss of Outer Membrane Integrity

**FIGURE 6:**
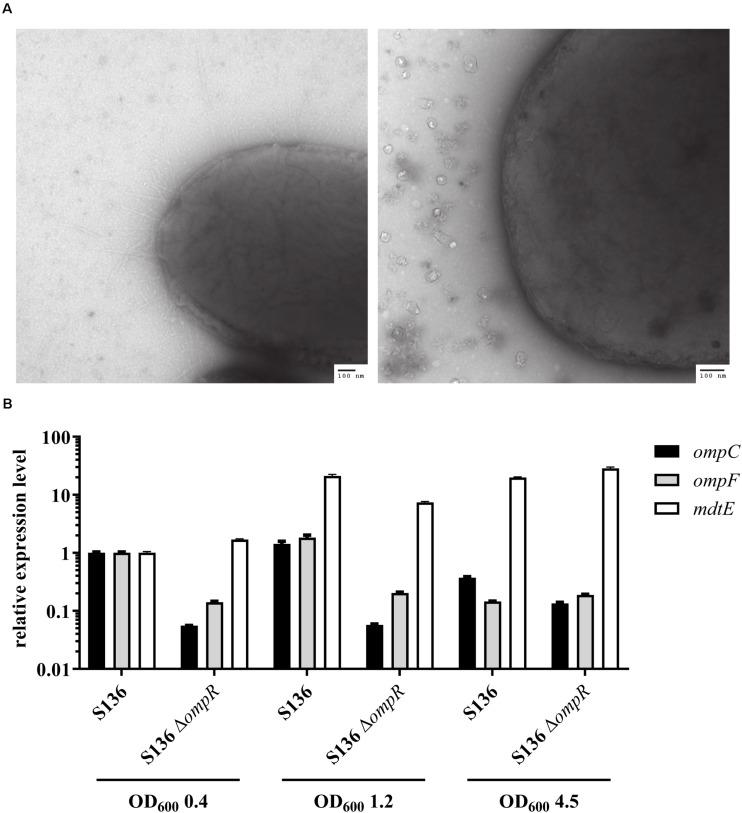
Investigation of potential OmpR-mediated DOC tolerance mechanisms. **(A)** Micrographs of AIEC S136 (left) and its related Δ*ompR* mutant (right) obtained by transmission electronic microscopy. **(B)** Expression levels of *ompC* (black bar), *ompF* (gray bar), and *mdtE* (white bar) were quantified by qRT-PCR in WT and Δ*ompR* S136 strains at mid-log (OD_600_ 0.4), late-log (OD_600_ 1.2), and late-stationary (OD_600_ 4.5) growth phases. The expression levels were normalized to the S136 WT strain at mid-log phase (means ± SEM, 2 technical replicates).

Antibiotics that do not cross the Gram-negative outer membrane were used to exclude that the inability to respond to DOC stress was caused by a non-specific loss of outer membrane integrity in the Δ*ompR* mutant strains. A 4-fold reduction in vancomycin MIC was observed in two of the eight Δ*ompR* mutant strains, while the MIC of rifampicin remained unchanged in all the strains (Supplementary Table 3). Moreover, the sensitivity to polymyxin B, a known membrane permeabilizing drug, did not increase in the Δ*ompR* mutants, indicating that the observed increased DOC sensitivity cannot be generalized to all membrane permeabilizing agents (Supplementary Table 2). Together, these data suggest that OmpR regulates a specific DOC tolerance mechanism in AIEC strains, as opposed to a non-specific loss of cell envelope integrity. This was confirmed using transmission electron microscopy excluding membrane defects on the Δ*ompR* AIEC mutants (Figure 6A).

Previous studies showed that downregulation of the outer membrane porin OmpF may be involved in adaptation to bile salt mediated stress (Thanassi et al., 1997). Since OmpR regulates the expression of outer membrane porins, we investigated whether OmpC and OmpF misregulation may be the driver of the observed OmpR-mediated DOC tolerance mechanism. Considering the complexity of OmpR-mediated regulation of porin expression (De la Cruz and Calva, 2010), we assessed the expression of *ompC* and *ompF* at three different growth phases, namely mid-log, late-log and late stationary phase. Both *ompC* and *ompF* genes were downregulated in the S136 Δ*ompR* mutant at all growth phases, indicating that the increased DOC sensitivity in the Δ*ompR* strains is not due to OmpC or OmpF upregulation (Figure 6B). In addition, the growth of the Δ*ompC*Δ*ompF* double mutant was not affected by DOC compared to the growth of the Δ*ompR* mutant (Supplementary Figure 5).

Besides porin downregulation, the expression of efflux pumps has been associated with increased resistance to DOC (Nishino and Yamaguchi, 2001). Interestingly, the MdtEF (previously known as YhiUV) efflux pump was shown to be moderately downregulated in a transcriptomic study performed on the *ompR* deleted *E. coli* K-12 MG1655 mutant (Seo et al., 2017), suggesting that MdtEF might play a role in OmpR-mediated DOC tolerance mechanism. We showed that *mdtE* expression is not regulated by OmpR in the S136 AIEC strain when tested at mid-log and late-stationary growth phases (Figure 6B). We observed a moderate downregulation (3-fold) of *mdtE* expression in the Δ*ompR* mutant compared to the WT strain when tested at late-log growth phase, however, *mdtE* remained overexpressed compared to mid-log growth phase. To note, DOC supplementation did not affect the level of *ompC*, *ompF* or *mdtE* expression at mid-log growth phase (Supplementary Figure 6).

## Discussion

Signal transduction systems, such as TCSs, are essential for bacteria to communicate and adapt within the harsh gut environment, ultimately leading to the modulation of their fitness and pathogenicity (Breland et al., 2017). The specific enrichment of AIEC strains in the intestinal ecosystem of CD patients is poorly understood (Kittana et al., 2019). The results from a previous study showed that OmpR is involved in *in vitro* adhesion and invasion capacities of the LF82 AIEC reference strain, suggesting that OmpR may contribute to AIEC-mediated exacerbation of CD (Rolhion et al., 2007).

In this study, we demonstrated the key role of OmpR in AIEC pathogenesis in CEABAC10 mouse infection model. However, we also showed that the role of OmpR in adhesion *in vitro* is not necessarily conserved in all AIEC strains, suggesting that other virulence mechanisms may be regulated by OmpR. We uncovered that OmpR is involved in AIEC tolerance toward the secondary bile salt DOC. DOC is present in large quantity in the gastrointestinal tract as it is produced by the microbiota from the conversion of the primary bile salt cholic acid (Ridlon et al., 2006; Pavlidis et al., 2015). This supports an important role for OmpR-mediated bile tolerance in AIEC survival and subsequent colonization of the gastrointestinal tract, ultimately leading to dysbiosis and enhanced chronic inflammation.

Bacterial cell-envelope structure maintenance and remodeling are required to resist to the action of bile salts, which is consistent with the role of the OmpR/EnvZ TCS in bile response (Merritt and Donaldson, 2009; Hernández et al., 2015; Sistrunk et al., 2016). Bile salts induce membrane damages that are likely to trigger OmpR activation through the envelope stress response pathway (Hews et al., 2019). Alternatively, it has been suggested that bile salts induce DNA supercoiling and it has been established that the OmpR/EnvZ TCS responds to DNA supercoiling, suggesting that OmpR/EnvZ may indirectly sense bile salts (Begley et al., 2005; Cameron and Dorman, 2012). It has been recently shown that OmpR from *Vibrio cholerae* is activated by bile salts (Kunkle et al., 2020). In contrast, using *ompC* and *ompF* expression levels as a surrogate for OmpR activation, we did not observe DOC-mediated activation of OmpR in AIEC. Another TCS from the OmpR family, namely PhoPQ, is required for *Salmonella spp.* to resist the action of bile (Velkinburgh and Gunn, 1999; Prouty and Gunn, 2000). Interestingly, as for OmpR in AIEC, PhoPQ conferred enhanced resistance preferentially toward DOC over other bile salts, suggesting that the two distinct TCSs regulate common DOC-induced stress response mechanisms in the two species. However, the exact mechanism by which PhoPQ enhances bile resistance in *Salmonella* remains to be elucidated (Villarreal et al., 2014).

We showed that loss of DOC tolerance was not due to loss of membrane integrity, suggesting that OmpR regulates a specific mechanism allowing AIEC to cope with DOC-induced stress. One of the DOC resistance mechanisms in *E. coli* consists in *micF*-mediated downregulation of OmpF porin synthesis to exclude charged molecules such as DOC from entering bacterial cells (Bernstein et al., 1999). Since OmpR is a known regulator of *micF* and *ompF* expression, we hypothesized that *ompF* downregulation might be the driver of OmpR-mediated DOC tolerance (Thanassi et al., 1997). However, we demonstrated that OmpR-mediated DOC tolerance in AIEC was not related to *ompF* porin downregulation. Another DOC resistance mechanism consists in the overexpression of specific efflux pumps (Nishino and Yamaguchi, 2001), among which the MdtEF was recently suggested to be regulated by OmpR in *E. coli* K-12 (Seo et al., 2017). However, we showed that the MdtEF efflux is only transiently regulated by OmpR resulting in limited downregulation of the efflux pump in the studied AIEC strain.

Alternatively, exposure to bile salts triggers a profound metabolic remodeling that favors intracellular accumulation of acetyl-CoA, ultimately conferring AIEC strains with a competitive advantage over commensal bacteria, including commensal *E. coli*, to colonize the gastrointestinal tract (Delmas et al., 2019). Interestingly, OmpR has been described as a key regulator of *E. coli* metabolism, with for instance the regulation of the GltA citrate synthase that controls the entry of acetyl-CoA into the tricarboxylic acid cycle (Chakraborty and Kenney, 2018). Therefore, an altered regulation of GltA expression in the *ompR* deleted AIEC mutants may be involved in the observed loss of bile salt tolerance and gut colonization defect of these strains. Further studies are needed to shed light on the OmpR-mediated bile salt tolerance mechanism.

Our work provides additional insights into the role of OmpR in AIEC pathogenesis. The newly discovered role of OmpR in AIEC DOC tolerance suggests that OmpR inhibition would interfere with different aspects of the AIEC virulence arsenal. Therefore, OmpR inhibition could be an alternative strategy for CD-treatment enabling a limited impact on commensal gut microbiota compared to standard antimicrobial treatments.

## Data Availability Statement

The original contributions presented in the study are included in the manuscript/Supplementary Materials, further inquiries can be directed to the corresponding author/s.

## Ethics Statement

The animal study was reviewed and approved by The Committee for Research and Ethical Issues of the Department of Auvergne (CEMEA Auvergne; Permit Number: CEMEAA, 2018103015295515).

## Author Contributions

VL: investigation, methodology, validation, formal analysis, visualization, and writing – original draft. AS: investigation, methodology, validation, formal analysis, visualization, and writing – review and editing. MP, MG, and SL: conceptualization, funding acquisition, and writing – review and editing. NB: conceptualization, project administration, funding acquisition, supervision, and writing – review and editing. CK: conceptualization, project administration, funding acquisition, supervision, visualization, and writing – review and editing. VT: conceptualization, project administration, supervision, formal analysis, visualization, writing – original draft, and writing review and editing. All authors contributed to the article and approved the submitted version.

## Conflict of Interest

MP, MG, SL, CK, and VT own equity in BioVersys. The remaining authors declare that the research was conducted in the absence of any commercial or financial relationships that could be construed as a potential conflict of interest.
