# Supplementary material for: The Role of OmpR in Bile Tolerance and Pathogenesis of Adherent-Invasive Escherichia coli
Source: Front Microbiol. 2021 Jun 28;12:684473. doi: 10.3389/fmicb.2021.684473 (PMC8273539; doi:10.3389/fmicb.2021.684473)
Supplement: Supplementary file 1 [file Data_Sheet_1.pdf]

Supplementary material

Table S1: List of primers

Name	Sequence	Description
**Genetic engineering**		
***ompR* deletion / complementation**		
oCK647	gaggaattcgagctcggtaccCGGTTTCACGTACTCGATAGC	*ompR*-up F
oCK648	tcgcctcatgcccgggGTTTATACTCCCAAAGGTTCG	*ompR*-up R
oCK649	aaaccccgggCATGAGGCGATTGCGCTTC	*ompR*-down F
oCK650	ctatcaacaggagtccaagactagtATCCGCCAGTTGCTTAACACC	*ompR*-down R
oCK354	CCGAGCGTTCTGAACAAATC	*plasmid integration*
oCK576	CATCGGCAGGAGGTTAAGAC	*ompR* deletion verification F
oCK578	ATCAGCAGCGTGCGGTCATC	*ompR* deletion verification R
oVT559	gctcggtacccggggatcctctagaaagaggagaaaATGCAAGAGAACTACAAGATTC	OmpR complementation F
oVT361	cgcaagcttgcatgcctgcagATAAGTCGTCACCAGGCTG	OmpR complementation R
**qRT-PCR**		
oVT614	TGCAGTTTCCAGCTCCAAAC	*ompC*-F
oVT615	AGCGTCGTATTTCAGACCAC	*ompC*- R
oVT436	AAAAACGAGCGTGACACTGC	*ompF*- F
oVT437	AGCACCAACGATACCAAAGC	*ompF* R
oBV97	TGATGCTGGCTGAAAACACC	*rpoD*- F
oBV98	TTCAACGGTGCCCATTTCAC	*rpoD*- R
**Sequencing**		
oVT357	AGGGGCGTTTTCATCTCGTT	*ompR-F*
oVT599	TCGGCAAAATCGCGAAGTTC	*ompR-R*
oVT1083	GTAAGCTGACGAACCAGGCA	*fim-F*
oVT1084	TAATCTCTGGCTCCCGTTGC	*fim-F*

Table S2: MIC of standard of care antibiotics against WT and Δ*ompR* AIEC strains

Strain		MIC (µg/ml)									
		IPM	PMB	CIP	MIN	KAN	GEN	AMK	TOB	CHL	SPT
MG1655	WT	0.0625	0.25	<0.008	1	1	0.125	0.5	0.25	4	8
	Δ*ompR*	0.031	0.25	0.008	1	1	0.125	0.5	0.125	8	8
7136	WT	0.0625	0.25	0.008	0.5	2	0.5	1	0.5	4	8
	Δ*ompR*	0.031	0.25	0.008	0.5	2	0.25	1	0.25	4	8
S136	WT	0.0625	0.25	0.016	2	2	0.5	1	**1**	8	16
	Δ*ompR*	0.031	0.25	0.008	2	2	0.5	1	**0.25**	8	16
S179	WT	0.0625	0.25	0.008	0.5	4	0.5	1	0.5	8	32
	Δ*ompR*	0.0625	0.25	0.008	0.5	2	0.5	1	0.5	8	16
S135	WT	0.0625	**0.25**	0.008	0.5	2	0.5	2	0.5	4	8
	Δ*ompR*	0.031	**1**	0.016	0.5	4	0.25	1	0.5	4	16
S244	WT	0.0625	0.25	0.008	0.5	2	0.5	**2**	0.5	4	16
	Δ*ompR*	0.031	0.5	0.008	0.5	2	0.25	**0.5**	0.25	8	16
S52	WT	0.125	0.25	**<0.008**	1	4	1	2	1	4	16
	Δ*ompR*	0.125	0.25	**0.016**	1	4	1	2	1	8	16
S162	WT	0.031	0.5	<0.008	0.5	4	0.5	1	0.5	4	16
	Δ*ompR*	0.031	0.5	0.008	0.5	2	0.5	1	0.5	8	32
LF31	WT	0.0625	0.5	<0.008	**2**	**8**	0.5	2	0.5	4	16
	Δ*ompR*	0.031	0.25	<0.008	**0.5**	**2**	0.25	1	0.25	4	16

IPM: imipenem, PMB: polymyxin B, CIP: ciprofloxacin, MIN: minocycline, KAN: kanamycin, GEN: gentamycin, AMK: amikacin, TOB: tobramycin, CHL: chloramphenicol, SPT: spectinomycin. MIC shifts equal or higher than 4-fold between WT and Δ*ompR* strains are highlighted in bold.

Table S3: MIC of vancomycin and rifampicin against WT and Δ*ompR* AIEC strains

Strain		MIC (µg/ml)	
		Vancomycin	Rifampicin
MG1655	WT	128	4
	Δ*ompR*	64	2
7136	WT	128	4
	Δ*ompR*	128	4
S136	WT	128	2
	Δ*ompR*	64	2
S179	WT	128	4
	Δ*ompR*	64	4
S135	WT	**>128**	2
	Δ*ompR*	**64**	2
S244	WT	128	2
	Δ*ompR*	64	2
S52	WT	>128	8
	Δ*ompR*	>128	4
S162	WT	128	4
	Δ*ompR*	64	2
LF31	WT	**>128**	2
	Δ*ompR*	**64**	2

MIC shifts equal or higher than 4-fold between WT and Δ*ompR* strains are highlighted in bold.

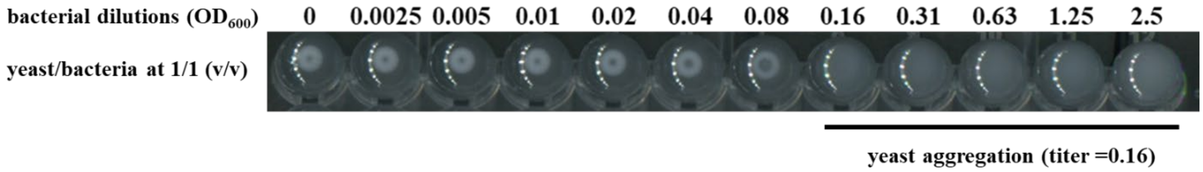

Figure S1: Example of a yeast aggregation titer assessment. A fixed amount of yeast cells suspension and decreasing concentrations of bacteria were mixed, and the lowest bacterial dilution still able to form homogenous aggregation was used as read-out. In this example, the yeast aggregation titer is 0.16.

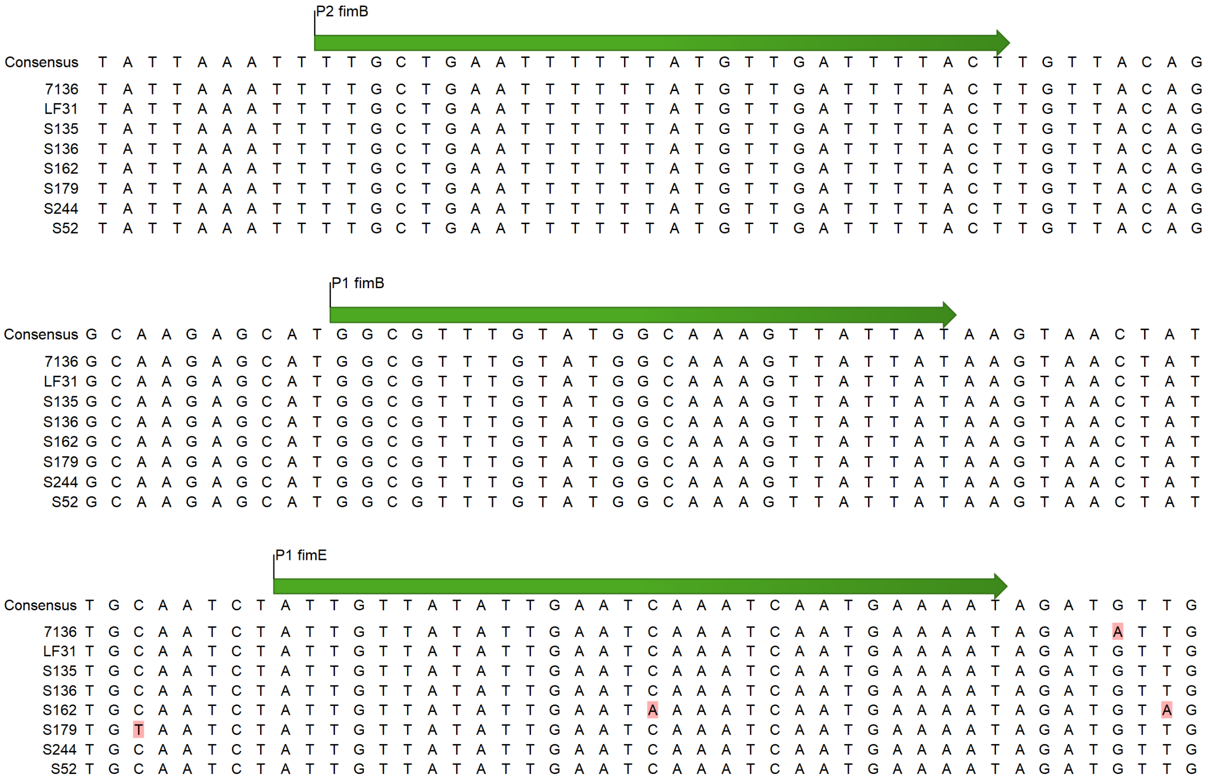

Figure S2: Sequence of the *fimB* and *fimE* promoters of the 8 AIEC strains. No mutations were found in the two promoters of *fimB* while the S162 AIEC strain carries a mutation in the *fimE* promoter.

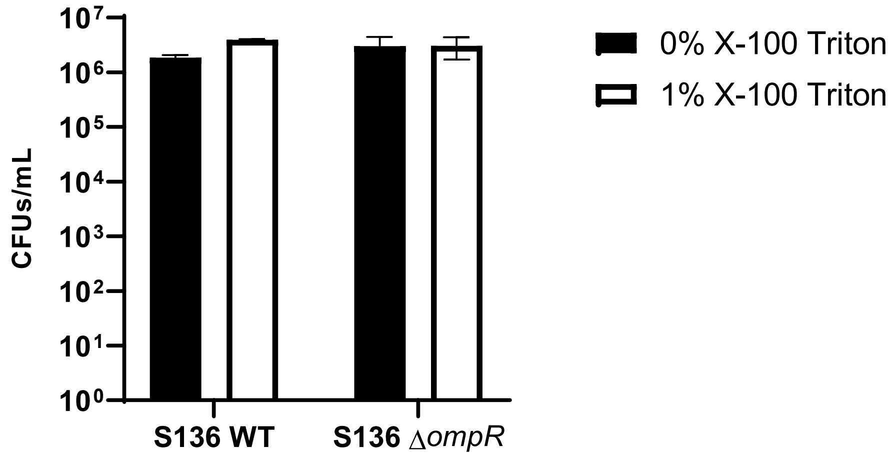

Figure S3: Effect of X-100 Triton on bacterial cells. The T84 cell lysis treatment (1% X-100 Triton for 5 min at room temperature) used in the adhesion assay was applied to the AIEC S136 WT and Δ*ompR* mutant to ensure that bacterial cells are not lysed.

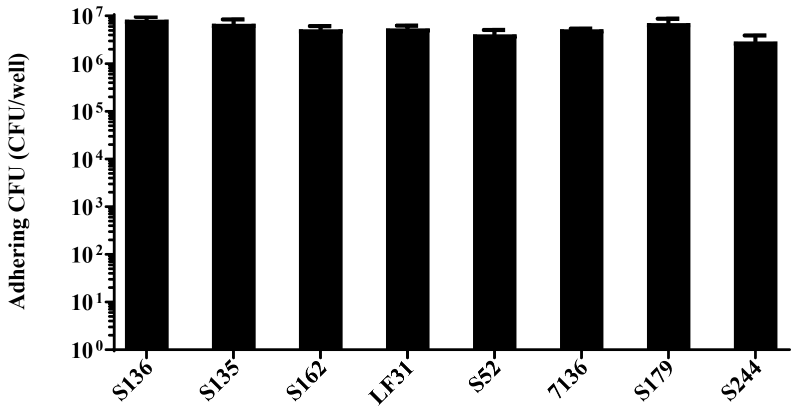

Figure S4: Adhesion levels of AIEC WT strains to intestinal epithelial cells T84.

Adhesion assay was performed with T84 intestinal epithelial cells infected with the different WT strains at a MOI of 10 bacteria/cell for 3 hrs. Results are expressed in CFU/well (means ± sem, 5 independent experiments).

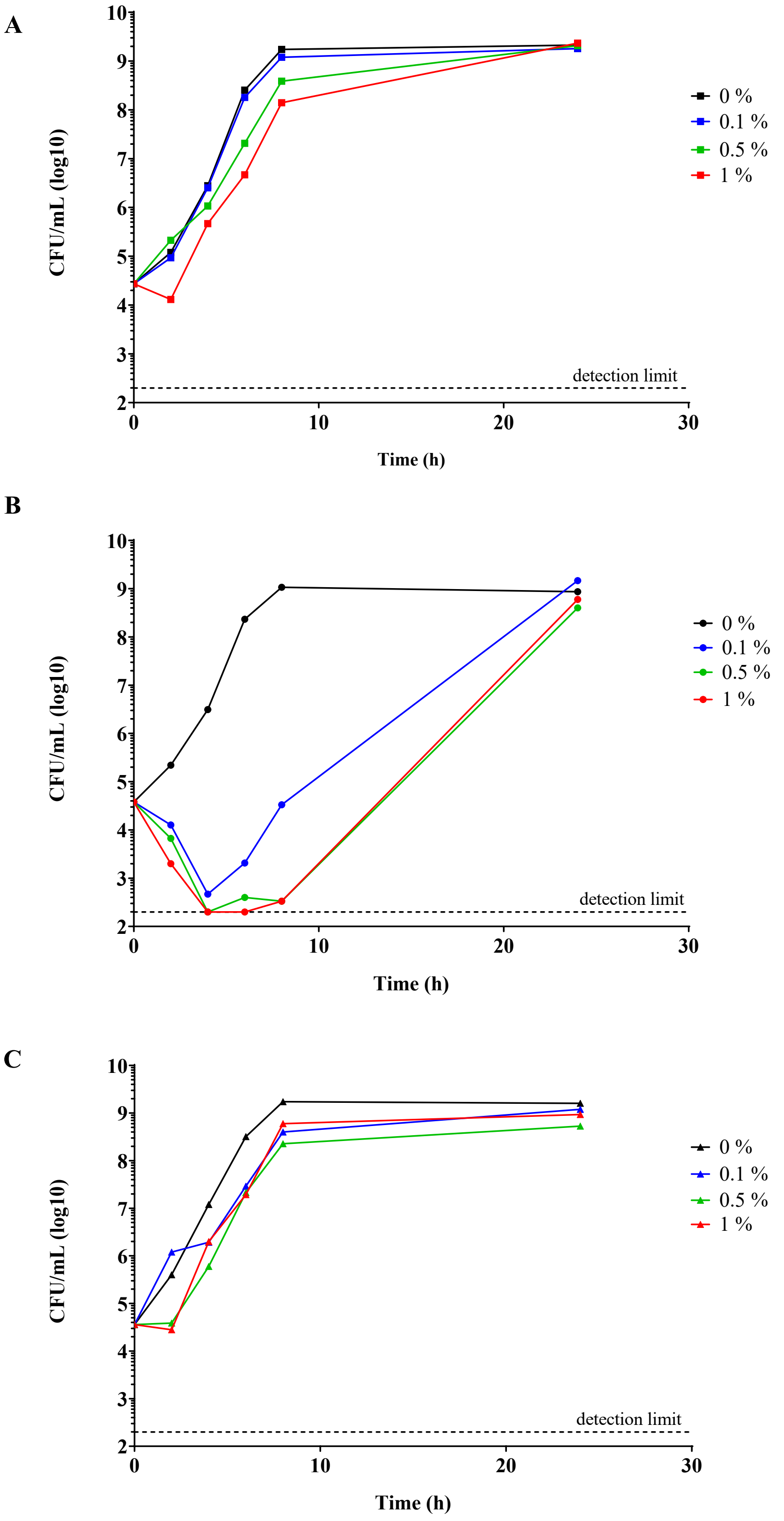

Figure S5: Growth curve of WT, Δ*ompR* and Δ*ompC*Δ*ompF* strains in presence of 0%, 0.1%, 0.5% and 1% of DOC.

The WT K12 MG1655 *E.coli* strain (A), its Δ*ompR* mutant (B) and Δ*ompC*Δ*ompF* (C) were grown in LB supplemented with (black line) 0 % DOC, (blue line) 0.1% DOC, (green line) 0.5% DOC and (red line) 1% DOC. Data representative of at least two independent experiments.

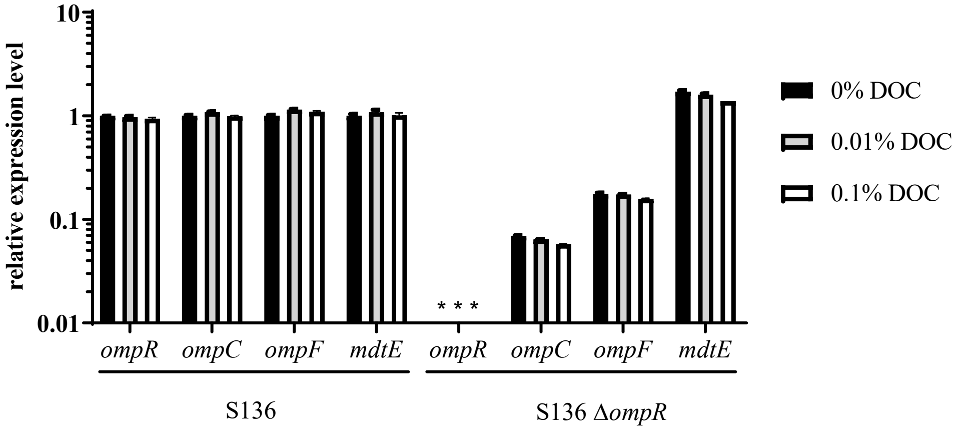

Figure S6: Influence of deoxycholate on gene expression.

Expression levels of *ompR*, *ompC, ompF* and *mdtE* were quantified by qRT-PCR in WT and Δ*ompR* S136 strains that were grown to mid-log growth phase (OD_{600} 0.4) and incubated with 0% (black), 0.01% (grey) and 0.1% (white) deoxycholate (DOC) for 30 minutes. The expression levels were normalized to the S136 WT strain without DOC (means ± sem, 2 technical replicates). * expression not detected.
